# No difference in dose distribution in organs at risk in postmastectomy radiotherapy with or without breast implant reconstruction

**DOI:** 10.1186/1748-717X-9-14

**Published:** 2014-01-09

**Authors:** Annelie Liljegren, Dmytro Unukovych, Giovanna Gagliardi, Judith Bjöhle, Marie Wickman, Hemming Johansson, Kerstin Sandelin

**Affiliations:** 1 Department of Molecular Medicine and Surgery, Karolinska Institute, Stockholm, Sweden; 2 Department of Breast and Endocrine Surgery, Karolinska University Hospital, Stockholm, Sweden; 3 Department of Oncology-Pathology, Karolinska Institute, Stockholm, Sweden; 4 Department of Reconstructive Plastic Surgery, Karolinska University Hospital, Stockholm, Sweden; 5 Department of Oncology, Karolinska University Hospital, Stockholm, Sweden; 6 Section of Radiotherapy Physics and Engineering, Medical Physics Department, Karolinska University Hospital, Stockholm, Sweden; 7 Karolinska University Hospital, R8:00, 17176 Stockholm, Sweden

## Abstract

The aim of this study was to quantify the variation in doses to organs at risk (ipsilateral lung and heart) and the clinical target volume (CTV) in the presence of breast implants. In this retrospective cohort study, patients were identified through the National Breast Cancer Register. Consecutive breast cancer patients undergoing mastectomy between 2009 and 2011 and completing a full course of postmastectomy radiotherapy (PMRT) were eligible. All included patients (n = 818) were identified in the ARIA© oncology information system and further stratified for immediate breast reconstruction (IBR+, n = 162) and no immediate breast reconstruction (IBR-, n = 656). Dose statistics for ipsilateral lung, heart and CTV were retrieved from the system. Radiation plans for patients with chest wall (CW) only (n = 242) and CW plus lymph nodes (n = 576) irradiation were studied separately.

The outcome variables were dichotomized as follows: lung, V_20Gy_ ≤ 30% vs. V_20Gy_ > 30%; heart, D_mean_ ≤ 5 Gy vs. D_mean_ > 5 Gy; CTV, V_95%_ ≥ median vs. V_95%_ < median.

In the univariate and multivariate regression models no correlation between potential confounders (i.e. breast reconstruction, side of PMRT, CW index) and the outcome variables was found. Multivariate analysis of CW plus lymph nodes radiation plans, for example, showed no association of breast reconstruction with dosimetric outcomes in neither lung nor heart- lung V_20Gy_ (odds ratio [OR]: 0.6, 95%CI, 0.4 to 1.0, p = 0.07) or heart D_mean_ (OR: 1.2, 95%CI, 0.5 to 3.1, p = 0.72), respectively.

CTV was statistically significantly larger in the IBR+ group (i.e. included breast implant), but no correlation between the implant type and dosimetric characteristics of the organs at risk was revealed.

In the current study, the presence of breast implants during postmastectomy radiotherapy was not associated with increased doses to ipsilateral lung and heart, but CTV definition and its dosimetric characteristics urge further evaluation.

## Background

Postmastectomy radiotherapy (PMRT) is shown to reduce the risk of local recurrence and overall mortality in patients with node-positive breast cancer [1]. Recent evidence that radiotherapy (RT) is beneficial for patients with one to three involved nodes or with high-risk node-negative disease has extended the application of PMRT [2,3].

Women operated on with total mastectomy are potential candidates for a breast reconstruction [4]. The numbers of immediate breast reconstructions (IBRs) have increased steadily [5,6] with the predominance of the implant based techniques today [7].

In three dimensional computer-tomography based (3DCT) PMRT planning, among all the OARs, heart and lungs are the structures most difficult to protect. RT was shown to cause a variety of radiation-induced changes in heart (e.g. coronary artery disease, cardiomyopathy, conduction disorders) and the risk of coronary events linearly increases with the mean dose to the heart [8,9]. One of the most common RT-induced reactions in lung is radiation pneumonitis that correlates with the dose distribution in the lung [10,11]. The presence of an implant implies a displacement of soft tissues within the target volume and may potentially increase lung and heart irradiation [12].

Two recent studies from the same institution concluded that excellent chest wall radiation coverage, local control and acceptable doses to risk organs could be achieved in the presence of breast implants during intensity modulated radiation therapy (IMRT) [13,14].

To our knowledge, no studies have assessed the impact of breast implants on dose distribution in a large cohort of patients after mastectomy undergoing conventional tangential radiotherapy*.*

The aim of this study was to quantify the variation in doses to organs at risk (ipsilateral lung and heart) and the target volume in the presence of breast implants.

## Patients and methods

### Study population

The Swedish National Breast Cancer Register was used for patient identification, and all women diagnosed with breast cancer and operated on with total mastectomy within the Stockholm-Gotland area between January 1 2009 and December 31 2011 and receiving postmastectomy radiotherapy were eligible. Data on type and date of breast cancer surgery, tumor characteristics (laterality, size and lymph nodes) and planned treatment characteristics (radiotherapy, chemotherapy, endocrine therapy, and immunotherapy) were obtained from the registry. Subsequently, these patients were identified in the ARIA© (Varian Medical Systems, Palo Alto, California)- an oncology information system prospectively maintained at two RT units at Karolinska University Hospital. Individual information on dates of radiotherapy (start and end), target volume (chest wall or chest wall plus lymph nodes), total dose, number of fractions, boost and bolus (if any), were retrieved from the RT charts in ARIA©.

The inclusion criteria were patients undergoing mastectomy as their primary surgery for breast cancer and completing a full course of PMRT to ipsilateral chest wall +/- regional lymph nodes.

**Figure 1:**
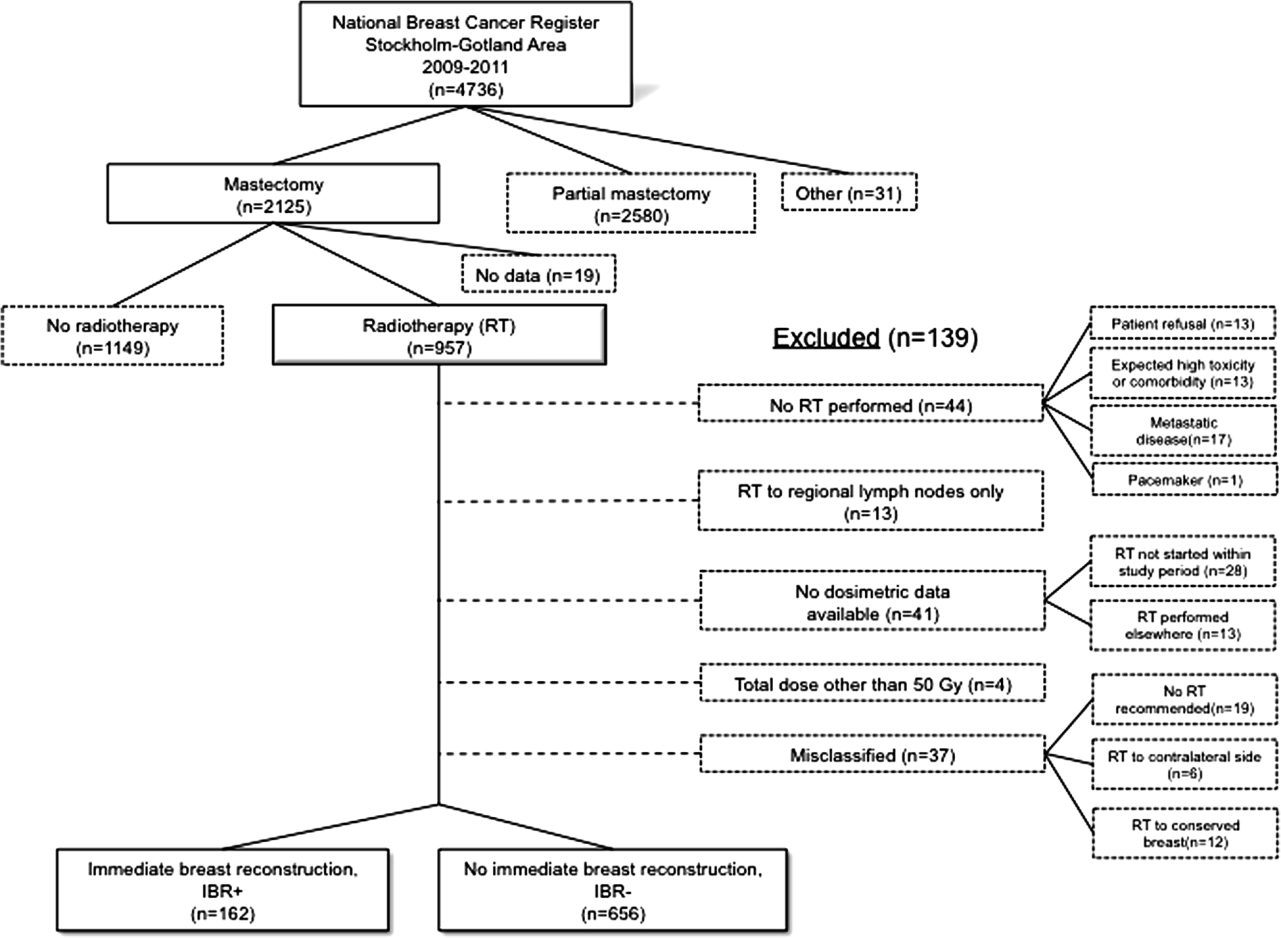
Study population, eligibility and inclusion/exclusion.

Patients were excluded for the following reasons (Figure 1):

1) No radiotherapy performed *de facto*.

2) Radiotherapy to other than ipsilateral chest wall or chest wall plus lymph nodes target area as some patients may have received RT to supraclavicular and/or axillary lymph nodes only.

3) No dosimetric data available in the verification system, *i.e.* RT was given elsewhere or has not started within the study period.

4) Radiotherapy course with a fractionation different than 2 Gy in 25 fractions.

5) Misclassified patients, *e.g.* treated with breast conservation therapy or those receiving RT to contralateral side, or when changes in adjuvant treatment plan ruled out PMRT.

### Target volume definition and radiotherapy technique and planning

All patients received full course of RT at one of the units, either at the Karolinska University Hospital in Solna (n = 425) or at the Southern Hospital (n = 393), Stockholm, Sweden. When CW only was included in the CTV the treatment technique consisted of two tangential fields. For those cases where the CTV included the lymph nodes, an isocentric technique was used, consisting of tangential fields covering CW and usually three fields covering the lymph nodes in the supraclavicular fossa and in the axillary regions. According to the institution’s local practice, internal mammary nodes (IMNs) were not specifically targeted. Both in the treatment of the chest wall and in the treatment of the chest wall plus lymph nodes field-in-field solutions were applied where necessary [15].

Conventional tangential external-beam radiotherapy with 6-MV photons was used in all cases, some patients also received additional 15-MV (n = 56, 6.8%) or 18-MV (n = 21, 2.6%) photon fields. Total prescribed dose was 50 Gy in 2 Gy daily fractions. Additional boost dose to the mastectomy scar, bolus, or a combination was utilized in 24 (3.0%), 31 (3.9%), and 3 (0.4%) cases, respectively.

3D CT-based radiation treatment planning was performed using the Varian Medical System Platform software (Varian Medical Systems Inc., Palo Alto, USA). Ipsilateral lung was contoured using auto-outline tool, whereas heart and clinical target volume (CTV) were delineated manually according to the RTOG guidelines. CTV was defined as chest wall only (CW) for local radiotherapy plans or CW plus lymph nodes (*i.e.* axillary, infraclavicular and supraclavicular) for loco-regional radiotherapy. In patients with IBR, CTV was always delineated comprising the breast implant. Planning target volume was defined by adding 5-7 mm margin around CTV. Clinical protocols for radiotherapy planning were the same during the study period.

Dose calculations were performed with the Eclipse Treatment Planning System using the Analytical Anisotropic Algorithm, AAA (Varian). The dose was prescribed so that the CTV would be encompassed between 95% and 105% isodoses. According to the local protocols the constraints to the ipsilateral lung was V_20Gy_ (% of volume receiving 20 Gy) <30%. The constraints to the heart were mean dose < 5 Gy, V_25Gy_ <10%, and normal tissues complications probability (NTCP) <1% [16,17].

### Implant characteristics and localization

**Figure 2:**
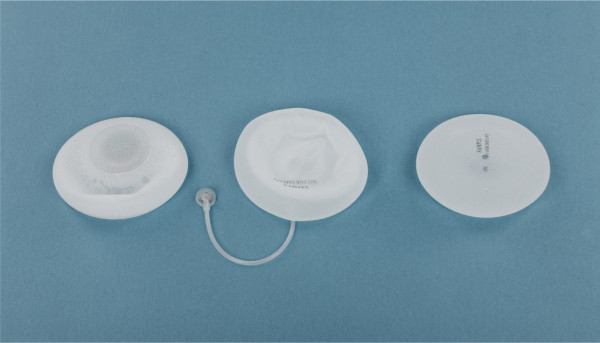
**Breast implants (*from left to right*): temporary expander (*type I*), expandable implant (*type II*), permanent implant (*type III*)**.

**Figure 3:**
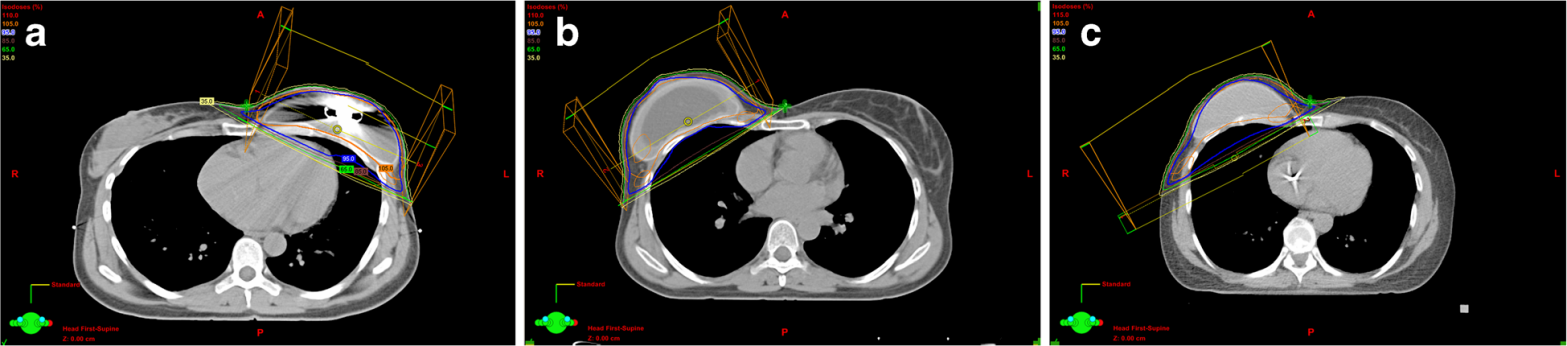
**Radiotherapy plan with: A. Temporary expander with magnet (*type I*), B. Expandable implant (*type II)*, C. Permanent implant (*type III*).**

Three types of implants were used during the study period (Figure 2 and Figure 3): *type I*, temporary expanders with a single lumen and an integrated magnetic port at the center of the implant for postoperative expansion; *type II*, expandable implants that are designed for a definitive one-stage breast reconstruction. They contain an outer chamber with silicon gel and a smaller inner chamber that may be inflated postoperatively with saline via the remote injection port placed subcutaneously on the chest wall; *type III*, permanent implants filled with silicon gel with a predefined volume.

At the same operation as the mastectomies, the implants were placed under total muscular coverage on the chest wall. No acellular dermal matrices have been used during the study period. In patients with expandable implants, the gradual expansion was performed in several sessions taking into consideration covering tissues.

Currently we avoid using expanders with the integrated magnetic ports in patients that are likely to receive PMRT. Potential problems with the magnetic port include difficulties in imaging (Figure 3a) [18,19] and cause perturbation in dose distribution around the magnet [20-22]. In the current study, we did not focus on these issues and thus excluded this subgroup from the CTV dosimetric assessment. An experimental study on this particular type of implants will be reported separately.

### Data collection and variables definition

The data obtained from the Swedish National Breast Cancer Registry, i.e. laterality of cancer, laterality of RT, reconstruction vs. mastectomy alone, were additionally verified for each patient in ARIA.

Dose Volume Histograms (DVHs) as well as dose statistics for ipsilateral lung, heart, and CTV were retrieved from the system. Ipsilateral lung dosimetry was assessed using minimum, maximum and mean dose to the lung, as well as V_20Gy_. The heart dosimetry was evaluated only in patients with left-sided breast cancer due to the fact that irradiation of the heart in the right-sided plans was negligible. Heart variables included minimum, maximum, mean dose and V_25Gy_.

CTV coverage was defined as percentage of clinical target volume covered by ≥95% of isodose (CTV V_95%_). CTV V_95%_ was evaluated both in the absolute measures (cm^3^ covered with 95% of isodose) as well as the relative measures (% of CTV covered with 95% of isodose).

In order to take into account patients’ rib cage shape, the following parameters were also considered: internal transverse diameter (T), measured between the lateral-most points of the rib cage; anterioposterior diameter (AP), from the back of the sternum to the front of the vertebra; and hemithorax anterioposterior diameter, between the anterior-most point and posterior-most point of the ipsilateral index hemithorax. All measurements were performed at the mamillary slice of CT scans. Chest wall index (CWi) was defined as the ratio between the transverse and the antero-posterior diameter (T/AP) as suggested by Haller et al. [23].

### Statistical analysis

STATA/SE (Version 11.1) for PC and MacOS, StataCorp, TX, USA, was used for all statistical analyses. Pearson’s chi-square or *t*-test when appropriate were utilized for assessment of differences in patients with (IBR+) and without (IBR-) breast reconstruction.

Chest wall, CW (n = 242) and chest wall plus lymph nodes, CW + LN (n = 576) radiation plans were studied separately. For the purpose of statistical analysis, the outcome variables were dichotomized as follows: Lung: V_20Gy_ ≤ 30% vs. V_20Gy_ >30%; Heart: D_mean_ ≤5 Gy vs. D_mean_ > 5 Gy. In the assessment of heart avoidance, only left-sided RTPs were used. Univariate and multivariate regression models were performed to test the association of outcome variables and potential confounders (breast reconstruction, side of RT, CW index). The results were presented as odds ratios (OR) with 95%CI and *p*-value. Reported *p*-values from these models referred to the Wald-test. A two-tailed *p* < 0.05 was considered significant in all statistical tests.

### Ethical approval

This study was approved by the Regional Research Ethics Committee in Stockholm 2010/1242-31 and 2011/1861 32.

## Results

### Patients characteristics and planned treatment

From 957 patients who fulfilled the inclusion criteria, 138 women were excluded and the remaining 818 were included into the study (Figure 1). Table 1 summarizes patients’ characteristics stratified for immediate breast reconstruction (IBR+) and no immediate breast reconstruction (IBR-).

**Table 1:** Demographic and treatment characteristics for 818 patients undergoing mastectomy and receiving postmastectomy radiotherapy

	**Total**	**IBR+**	**IBR-**	
Characteristics	n = 818 (%)	n = 162 (%)	n = 656 (%)	*P-*value¶
Age at mastectomy, years				
Median [min-max]	58 [21-90]	45 [21-69]	58 [21-90]	<0.001
≤55	363 (44.4)	136 (84.0)	227 (34.6)	
>55	455 (55.6)	26 (16.0)	429 (65.4)	<0.001
Calendar year mastectomy				
2009	260 (31.8)	61 (37.7)	199 (30.3)	
2010	292 (35.7)	47 (29.0)	245 (37.4)	
2011	266 (32.5)	54 (33.3)	212 (32.3)	0.093
Side^#^				
Right	391 (47.8)	90 (55.6)	301 (45.9)	
Left	427 (52.2)	72 (44.4)	355 (54.1)	0.027
Tumor size				
pT1	309 (37.8)	72 (44.4)	237 (36.1)	
pT2	333 (40.7)	57 (35.2)	276 (42.1)	
pT3	125 (15.3)	25 (15.4)	100 (15.2)	0.15
Missing*	51 (6.2)	8 (4.9)	43 (6.6)	
Lymph nodes				
pN0	295 (36.1)	83 (51.2)	212 (32.3)	
pN+	511 (62.5)	77 (47.5)	434 (66.2)	<0.001
Missing*	12 (1.5)	2 (1.2)	10 (1.5)	
Adjuvant chemotherapy				
Yes	388 (47.4)	91 (56.2)	297 (45.3)	
No	415 (50.7)	70 (43.2)	345 (52.6)	0.020
Missing*	15 (1.8)	1 (0.6)	14 (2.1)	
Neoadjuvant chemotherapy				
No	559 (68.3)	130 (80.2)	429 (65.4)	
Yes	259 (31.7)	32 (19.8)	227 (34.6)	<0.001
Endocrine therapy				
Yes	643 (78.6)	110 (67.9)	533 (81.3)	
No	164 (20.1)	49 (30.2)	115 (17.5)	<0.001
Missing*	11 (1.3)	3 (1.9)	8 (1.2)	
RT target				
Chest wall	242 (29.6)	80 (49.4)	162 (24.7)	
Chest wall plus lymph nodes	576 (70.4)	82 (50.6)	494 (75.3)	<0.001
Boost dose				
Yes	27 (3.3)	3 (1.9)	24 (3.7)	
No	791 (96.7)	159 (98.1)	632 (96.3)	0.33
Bolus field				
Yes	34 (4.2)	3 (1.9)	31 (4.7)	
No	784 (95.8)	159 (98.1)	625 (95.3)	0.12
Breast implant				
Permanent expander	92 (56.8)	92 (56.8)	-	
Permanent implant	40 (24.7)	40 (24.7)	-	
Temporary expander with a magnetic port	30 (18.5)	30 (18.5)	-	-

IBR indicates immediate breast reconstruction; RT, radiotherapy; pT, tumor size.

*Not included into statistical analysis.

#Including eleven patients with bilateral radiotherapy: IBR + (n = 2) and IBR- (n = 9).

¶Pearson *χ*^**2**^ test.

Patients’ mean age at the time of mastectomy was 46 years (SD = 8.5) in IBR+ and 58 years (SD = 13.6) in IBR- subgroups (p < 0.001). In the IBR+ group, patients were younger (<55 years: 84.0% vs. 34.6%, p < 0.001), neoadjuvant chemotherapy was given less frequently (19.8% vs. 34.6%, p < 0.001).

The IBR+ subgroup had significantly higher rates of negative lymph nodes (pN0: 51.2% vs. 32.3%, p < 0.001) and consequently received RT to regional lymph nodes less frequently (50.6% vs. 75.3%, p < 0.001). In addition, this subgroup was treated with adjuvant chemotherapy and endocrine therapy less frequently (45.3% vs. 56.2%, p = 0.02 and 67.9% vs. 81.3%, p < 0.001, respectively).

### Risk organs and clinical target volume dosimetric evaluation

Dosimetric characteristics of ipsilateral lung stratified for radiotherapy plan are presented in Table 2*.* In the chest wall subset, lung maximum dose was higher in IBR+ group (52.0 vs. 51.4 Gy, p = 0.002).

**Table 2:** Dosimetric and anthropometric characteristics* of ipsilateral lung and heart (n = 818)

	**Chest wall**				**Chest wall plus lymph nodes**			
Characteristics	Total n = 242	IBR+ n = 80	IBR- n = 162	*P*-value¶	Total n = 576	IBR+ n = 82	IBR- n = 494	*P*-value¶
Ipsilateral lung								
Volume, cm^3^	1431.2 [330.2]	1458.6 [318.8]	1417.0 [335.8]	0.36	1369.6 [362.2]	1382.6 [407.7]	1367.4 [354.5]	0.72
Mean dose, Gy	8.9 [3.8]	8.9 [5.2]	8.9 [2.8]	0.96	14.3 [2.1]	13.8 [2.3]	14.3 [2.1]	0.05
Minimum dose, Gy	0.3 [1.0]	0.4 [1.7]	0.2 [0.1]	0.11	0.2 [0.1]	0.3 [0.2]	0.2 [0.1]	<0.001
Maximum dose, Gy	51.6 [1.3]	52.0 [1.3]	51.4 [1.3]	0.002	51.8 [1.1]	51.5 [1.5]	51.8 [1.1]	0.06
V_20Gy_, %	16.4 [6.1]	15.8 [6.0]	16.7 [6.2]	0.30	28.7 [5.3]	28.1 [5.7]	28.8 [5.2]	0.22
Heart^‡^	*n = 118*	*n = 35*	*n = 83*		*n = 309*	*n = 37*	*n = 272*	
Volume, cm^3^	512.2 [135]	515.9 [115.5]	510.6 [143.0]	0.85	532.2 [117.8]	572.9 [143.1]	526.6 [112.4]	0.024
Mean dose, Gy	3.3 [1.9]	3.0 [0.9]	3.4 [2.1]	0.32	3.5 [1.5]	3.8 [1.2]	3.5 [1.5]	0.29
Minimum dose, Gy	0.2 [0.1]	0.2 [0.1]	0.2 [0.1]	0.25	0.3 [0.2]	0.3 [0.02]	0.3 [0.01]	0.27
Maximum dose, Gy	48.5 [5.5]	48.7 [5.6]	48.4 [5.4]	0.81	47.4 [7.2]	48.5 [5.2]	47.3 [7.5]	0.35
V_25Gy_, %	3.7 [2.4]	3.1 [1.7]	4.0 [2.7]	0.07	3.8 [2.6]	3.8 [2.1]	3.8 [2.7]	1.0

*All numbers in the rows indicate mean values [standard deviation].

V_20Gy_ indicates volume of the ipsilateral lung irradiated with 20 Gy; V_25Gy_, volume of heart irradiated with 25 Gy.

‡Calculated for left-sided plans only.

¶Student’s independent *t*-test.

In the CW + LN subset, IBR+ patients were significantly different from IBR- with regards to lung mean dose (13.8 vs. 14.3 Gy, p = 0.05) and lung minimum dose (0.3 vs. 0.2 Gy, p < 0.001).

Heart dosimetry was obtained for 72 IBR+ and 355 IBR- patients with left-sided tumors. No statistically significant differences in heart dosimetric characteristics such as heart V_25Gy_ or mean dose were identified between the groups (Table 2).

CTV definition in patients with IBR always included implant that makes the direct dosimetric comparisons between the groups inaccurate, *i.e.* CTV was larger in IBR+ compared to IBR- for both CW (629.4 vs. 458.4 cm^3^, p < 0.001) and CW + LN (1074.7 vs. 787.9 cm^3^, p < 0.001) radiation plans (Table 3). There was a difference in the rib cage shape and in the chest wall index between the groups (Table 3).

**Table 3:** Dosimetric and anthropometric characteristics* of clinical target volume and rib cage (n = 788)

	**Chest wall**			**Chest wall plus lymph nodes**		
Characteristics	IBR+ n = 59	IBR- n = 162	*P*-value¶	IBR+ n = 73	IBR- n = 73	*P*-value¶
CTV						
Volume, cm^3^	629.4 [283.4]	458.4 [273.6]	<0.001	1074.7 [263.0]	787.9 [289.0]	<0.001
Mean dose, Gy	50.2 [0.9]	50.7 [4.1]	0.31	50.4 [0.6]	50.4 [0.6]	0.97
V_95%_, % ^a^	91.2 [5.3]	91.4 [8.4]	0.83	93.7 [3.2]	93.3 [4.4]	0.43
V_95%_, cm^3^^b^	572.2 [248.9]	417.0 [252.1]	<0.001	1006.7 [251.6]	733.9 [270.0]	<0.001
V_105%_, % ^a^	10.8 [8.2]	15.3 [12.1]	0.009	11.8 [6.6]	12.9 [6.1]	0.17
V_105%_, cm^3^^b^	61.0 [37.9]	65.8 [56.6]	0.55	128.7 [79.6]	104.8 [68.6]	0.007
Rib cage^†^						
Transverse diameter, cm	23.5 [1.2]	23.5 [1.5]	0.70	24.2 [1.6]	23.4 [1.5]	<0.001
Anterioposterior diameter, cm	9.5 [1.5]	10.3 [1.5]	<0.001	9.8 [1.3]	10.3 [1.5]	0.006
Ipsilateral internal diameter, cm	14.2 [1.3]	15.5 [1.6]	<0.001	14.6 [1.4]	15.3 [1.6]	<0.001
Chest wall index	2.5 [0.5]	2.3 [0.4]	<0.001	2.5 [0.4]	2.3 [0.4]	<0.001

*All numbers in the rows indicate mean values [standard deviation].

Radiotherapy plans of patients with temporary expanders containing magnetic ports (n = 30) were excluded from the analyses.

CTV indicates clinical target volume; V_95%_ and V_105%_, volume irradiated with 95% and 105% of isodose.

^a^Relative volume assessment.

^b^Absolute volume assessment.

¶Student's independent *t*-test.

†Measured at mammillary level tomography slide.

### Regression analyses

The univariate analyses revealed no association between breast implant reconstruction and ipsilateral lung or heart dosimetry (Table 4) in neither the chest wall nor chest wall plus lymph nodes subsets. Among the possible confounders, only chest wall index was associated with lung V_20Gy_ in CW + LN subset (OR: 1.6, 95%CI, 1.1 to 2.2, p = 0.008). This association remained statistically significant in multivariate regression adjusting for reconstruction and side (Table 5).

**Table 4:** Univariate analysis of lung and heart dosimetry and possible confounders in patients with chest wall only and chest wall plus lymph nodes irradiation (n = 818)

		**Chest wall, n = 242**								**Chest wall plus lymph nodes, n = 576**							
		Lung V_20Gy_				Heart D mean*				Lung V_20Gy_				Heart D mean*			
Confounders		≤30%	>30%	OR (95%CI)	*P-value*	≤5Gy	>5Gy	OR (95%CI)	*P-value*	≤30%	>30%	OR (95%CI)	*P-value*	≤5Gy	>5Gy	OR (95%CI)	*P-value*
Reconstruction	IBR-	155	7			75	8			293	201			236	36		
	IBR+	75	5	1.5 (0.5 to 4.8)	0.52	35	0	-^#^	-^#^	56	26	0.7 (0.4 to 1.1)	0.13	31	6	1.3 (0.5 to 3.3)	
Side	Right	118	6			-	-			165	102			-	-		
	Left	112	6	1.1 (0.3 to 3.4)	0.93	110	8	-	-	184	125	1.1 (0.8 to 1.5)	0.58	267	42	-	-
Chest wall index	CWi ≤ med	118	7			57	7			222	119			162	26		
	CWi > med	112	5	0.8 (0.2 to 2.4)	0.64	53	1	0.2 (0 to 1.3)	0.09	127	108	1.6 (1.1 to 2.2)	0.008	105	16	0.9 (0.5 to 1.9)	0.88

CTV indicates clinical target volume; IBR, immediate breast reconstruction; CWi, chest wall index; med, median.

Lung V_20Gy_, volume of lung tissue irradiated with 20 Gy (strata: ≤30% vs. >30%); Heart D mean, mean dose delivered to heart (strata: ≤5 Gy vs. >5 Gy);

CTV V_95%_, percent of clinical target volume irradiated with ≤95% of isodose (strata: ≤median vs. >median).

*Analyzed for patient with left-sided radiation plans (n = 118/242 and n = 309/576).

#No patients with implant breast reconstruction received >5 Gy to heart.

**Table 5:** Multivariate analysis of lung and heart dosimetry and possible confounders in patients with chest wall and chest wall plus lymph nodes radiotherapy (n = 818)

		**Chest wall**		**Chest wall plus lymph nodes**	
		**n = 242**		**n = 576**	
Outcome variable	Covariate	OR (95%CI)	*P*-value	OR (95%CI)	*P*-value
Lung V_20Gy_					
	IBR- vs. IBR+	1.7 (0.5 to 5.8)	0.41	0.6 (0.4 to 1.0)	0.07
	Right vs. Left	1.1 (0.3 to 3.4)	0.91	1.1 (0.8 to 1.5)	0.59
	CWi ≤ median vs. CWi > median	0.7 (0.2 to 2.2)	0.50	1.7 (1.2 to 2.3)	0.004
Heart Dmean*		n = 118*		n = 309*	
	IBR- vs. IBR+	-^#^	-	1.2 (0.5 to 3.1)	0.72
	Right vs. Left	-	-	-	-
	CWi ≤ median vs. CWi > median	0.2 (0.3 to 1.8)	0.16	0.9 (0.5 to 1.8)	0.82

*IBR*, immediate breast reconstruction; *CWi*, chest wall index; *OR*, odds ratio; *CI*, confidence interval.

Lung V_20Gy_, volume of ipsilateral lung tissue irradiated with 20 Gy (strata: ≤30% vs. >30%); Heart Dmean, mean dose delivered to heart (strata: ≤5 Gy vs. >5 Gy).

*Analyzed for patients with left-sided radiation plans.

#No patients with implant breast reconstruction received >5 Gy to heart.

### Type of implant

In the IBR+ subgroup analysis we found no correlation between the type of implants and the three outcome dosimetric variables (data not shown). Time from mastectomy to radiotherapy start among patients with temporary expanders, permanent expanders, permanent implants, and no reconstruction was 4.5, 4.1, 4.8, and 3.8 months, respectively with no statistically significant difference (data not shown).

## Discussion

In the current study, the presence of a breast implant during postmastectomy radiotherapy was not associated with increased doses to ipsilateral lung and heart.

Koutcher *et al.* were the first to analyze radiotherapy plans in 41 patients with expandable implants. They reported excellent local control and acceptable heart and lung doses; 73% patients had adequate chest wall coverage, lung V_20Gy_ in the majority of patients was <20% and mean heart dose was 2.8 Gy. This study, however, is limited by the small sample size and the absence of a control group of patients without breast reconstruction [13].

In another publication, radiation plans of a mixed patient population with autologous tissue reconstruction from the abdomen (n = 107) and autologous tissue reconstruction from the back +/- implants (n = 5) were compared with selected controls without IBR (n = 106) matched by calendar year, side and tumor stage. Using a novel non-validated scoring system, the authors concluded that more than half of the patients in the IBR+ group had their radiation plans impaired. The coverage of the chest wall and ipsilateral intramammary nodes, and dose distribution in the lung were not optimal. The authors also suggested that in patients with locally advanced breast cancer, an option for delayed breast reconstruction should be considered due to potential problems with radiation delivery [24].

A recent case-control study compared 196 patients having implant-based IBR with 51 matched individuals without IBR, concluding that having an implant was associated with lower doses to the lung and non-superior doses to the heart. Notably, the target volumes for IBR- plans were not delineated and all ipsilateral lung and heart structures were delineated for study purposes *de novo*. The interpretation of the study results is difficult due to the fact that different radiation techniques were utilized in the two disparate groups of IBR+ and IBR- patients [14].

Another study analyses the impact of inclusion/exclusion inframammary lymph nodes into CTV in a mix series of breast implants (n = 10) and autologous tissue reconstructions (n = 10) [25]. Adequate coverage of reconstructed breast was demonstrated, regardless of reconstruction type, laterality and IMN inclusion. This experimental study is however limited by twenty RTPs and may poses a selection bias, as it does not include any reference group of patients without breast reconstruction.

The above studies are not directly comparable with our study. Firstly, in the current observational cohort study, all consecutive patients receiving PMRT were included and stratified according to breast reconstruction at a later stage. Secondly, different from other studies radiotherapy treatment technique (conventional tangential external-beam radiotherapy with 6-MV photons) and different calculation algorithm (AAA) were used for all patients. Thirdly, our definition of target volume is different from others as we classified radiation plans into two subsets: chest wall only and chest wall plus lymph nodes. Finally, irradiation of intramammary nodes that has been shown to correlate with higher doses to risk organs [14,24] is negligible in our cohort due to the differences in guidelines and intramammary nodes targeting. This issue, therefore, was not specifically addressed in this study.

CTV definition in patients with breast implants is extremely relevant and will be addressed in the next study, where we evaluate dosimetric characteristics in the area outside the implant (“CTV *excluding* implant”) being particularly important for the local tumor control. Furthermore, there are areas irradiated with higher doses (i.e. V105%) that may have a local influence and affect cosmesis and morbidity. In the current study, these excess doses have been seen within the 10-15% of CTV, however they might be spread along the whole CTV. These hot spots should be mapped and evaluated as the possible confounders for negative outcomes of breast reconstruction.

When assessing possible implications of the implant type, no significant differences in the OARs dosimetric characteristics were observed between the three groups.

Interestingly, a difference in rib cage shape between IBR+ and IBR- groups was found. These differences are not clear and might be attributed to the age-related changes in the thorax structure [26]; the clinical application of these finding needs to be further explored. We also found statistically significant differences between IBR+ and IBR- in the lung mean and minimum dose (in CW + LN subset), as well as lung maximum dose (in CW subset). However, the clinical significance of these data is doubtful as these endpoint measures do not take into consideration the volumetric component, i.e. how large the irradiated lung volume is [10,11].

The strengths of the study include its cohort design and sample size; consecutive patients were undergoing PMRT at two radiotherapy units, where the same radiation techniques were utilized regardless breast reconstruction or not. The actual radiation plans and DVHs obtained from ARIA hospital-based radiotherapy system, have been used in the analyses. Assessing the role of breast implant in the multivariate analysis, we adjusted for other potential confounding factors, especially introducing the chest wall index to control for the difference in the shape of the rib cage.

A weakness of the study might lie in its retrospective nature as we had a possibility to exclude some patients from the analyses. We also refrained from CTV dosimetric evaluation in the current study due to the fact that delineated CTV in IBR+ group always included breast implant. Mixing these different non-comparable volumes (*i.e.* CTV_IBR-_ and CTV_IBR+_) into one regression model appeared to be inaccurate and seemed misleading.

In conclusion, current study did not reveal differences in dose distribution in organs at risk among patients receiving PMRT with or without breast implants. Dosimetric characteristics and definition of CTV in patients with implants urges further evaluation. Further studies specifically addressing consequences of implants on PMRT planning and delivery will shed light on oncologic safety.

## Competing interests

The authors declare that they have no competing interests.

## Authors’ contribution

DU and AL made substantial contributions to acquisition of data. AL, DU, GG, JB, MW, HJ, and KS have all made substantial contributions to the conception and design of this study and interpretation of data. DU and HJ performed the statistical analyses. All authors have been critically involved in the drafting and revising of this manuscript, and read and approved the final manuscript.

## Authors’ information

Liljegren Annelie and Unukovych Dmytro are Joint first authors.
